# Study protocol of a randomized controlled trial to assess safety of teleconsultation compared with face-to-face consultation: the ECASeT study

**DOI:** 10.1186/s13063-023-07679-1

**Published:** 2023-12-08

**Authors:** Alejandro Rodríguez-Molinero, Gerard Carot-Sans, Roser Escrig, Cristian Tebé, Jacobo Arce, Carlos Pérez-López, Silvia Ballesta, Guillermo Verdejo, Ángel Cedeño, Mar Riera-Pagespetit, Sofia Vivas-Angeles, Jose L. Alarcon, Itziar Navarro, Silvia Toro, Llorenç Mateo, Ana J. Torres, Gerard Delmás, Helena Camell, Antonio Chamero, Montse Gasol, Jordi Piera-Jiménez, ECASeT Research Group, David Benaiges, Lidia Tikhomirova, Vicenç Torrente, Jesús Marimón, David Saavedra, Violeta Menendez, Elisabet Franquet, Luis M. Sierra, María López-Diéguez, Nuria Rodríguez, Jessica Gonzales, Oscar Macho-Pérez, Sandra Huguet, Lucas Degano, Francisco Pineda, Javier Errando

**Affiliations:** 1 Àrea de Recerca, Consorci Sanitari de L’Alt Penedès I GarrafEspirall, Vilafranca del Penedès, 61 08720 Barcelona, Spain; 2 Catalan Health Service, Barcelona, Spain; 3 Digitalization for the Sustainability of the Healthcare System (DS3), IDIBELL, Barcelona, Spain; 4 Biostatistics Unit of the Bellvitge Biomedical Research Institute (IDIBELL), L’Hospitalet de Llobregat, Barcelona, Spain; 5 Urology Department, Consorci Sanitari de L’Alt Penedès I Garraf, Vilafranca del Penedès, Barcelona, Spain; 6 Endocrinology Department, Consorci Sanitari de L’Alt Penedès I Garraf, Vilafranca del Penedès, Barcelona, Spain; 7 Department of Internal Medicine, Consorci Sanitari de L’Alt Penedès I Garraf, Vilafranca del Penedès, Barcelona, Spain; 8 Gastroenterology Department, Consorci Sanitari de L’Alt Penedès I Garraf, Vilafranca del Penedès, Barcelona, Spain; 9 Geriatrics Department, Consorci Sanitari de L’Alt Penedès I Garraf, Vilafranca del Penedès, Barcelona, Spain; 10 Department of Surgery, Consorci Sanitari de L’Alt Penedès I Garraf, Vilafranca del Penedès, Barcelona, Spain; 11 Nefrology Department, Consorci Sanitari de L’Alt Penedès I Garraf, Vilafranca del Penedès, Barcelona, Spain; 12 Musculoskeletal Area, Consorci Sanitari de L’Alt Penedès I Garraf, Vilafranca del Penedès, Barcelona, Spain; 13 Maternal-Child Area, Consorci Sanitari de L’Alt Penedès I Garraf, Vilafranca del Penedès, Barcelona, Spain; 14 Innovation Department, Consorci Sanitari de L’Alt Penedès I Garraf, Vilafranca del Penedès, Barcelona, Spain; 15 Anesthesiology Department, Consorci Sanitari de L’Alt Penedès I Garraf, Vilafranca del Penedès, Barcelona, Spain; 16 Department of Pharmacology, Therapeutics, and Toxicology, Universitat Autònoma de Barcelona, Bellaterra, Spain; 17 Faculty of Informatics, Telecommunications and Multimedia, Universitat Oberta de Catalunya, Barcelona, Spain

**Keywords:** Remote consultation, Video consultation, Teleconsultation

## Abstract

**Background:**

The use of remote consultation modalities has exponentially grown in the past few years, particularly since the onset of the COVID-19 pandemic. Although a huge body of the literature has described the use of phone (tele) and video consultations, very few of the studies correspond to randomized controlled trials, and none of them has assessed the safety of these consultation modalities as the primary objective. The primary objective of this trial was to assess the safety of remote consultations (both video and teleconsultation) in the follow-up of patients in the hospital setting.

**Methods:**

Multicenter, randomized controlled trial being conducted in four centers of an administrative healthcare area in Catalonia (North-East Spain). Participants will be screened from all individuals, irrespective of age and sex, who require follow-up in outpatient consultations of any of the departments involved in the study. Eligibility criteria have been established based on the local guidelines for screening patients for remote consultation. Participants will be randomly allocated into one of the two study arms: conventional face-to-face consultation (control) and remote consultation, either teleconsultation or video consultation (intervention). Routine follow-up visits will be scheduled at a frequency determined by the physician based on the diagnostic and therapy of the baseline disease (the one triggering enrollment). The primary outcome will be the number of adverse reactions and complications related to the baseline disease. Secondary outcomes will include non-scheduled visits and hospitalizations, as well as usability features of remote consultations. All data will either be recorded in an electronic clinical report form or retrieved from local electronic health records. Based on the complications and adverse reaction rates reported in the literature, we established a target sample size of 1068 participants per arm. Recruitment started in May 2022 and is expected to end in May 2024.

**Discussion:**

The scarcity of precedents on the assessment of remote consultation modalities using randomized controlled designs challenges making design decisions, including recruitment, selection criteria, and outcome definition, which are discussed in the manuscript.

**Trial registration:**

NCT05094180. The items of the WHO checklist for trial registration are available in Additional file 1. Registered on 24 November 2021.

**Supplementary Information:**

The online version contains supplementary material available at 10.1186/s13063-023-07679-1.

## Roles and responsibilities

The ECASeT trial is sponsored by the Consorci Sanitari Alt Penedès—Garraf (CSAPG), a healthcare provider within the Catalan Health System, and funded by the Instituto de Salud Carlos III (ISCIII) through the project “PI22/01056” (co-funded by the European Union). Neither the sponsor nor the funder agency contributed to the trial design; they are not involved in trial conduct and will not be involved in the analysis of the results.

The scientific committee, formed by AR-M, GC-S, RE, CT, JA, CP-L, AT, GD, ACh, MG, and JP-J, was responsible for key decisions regarding the primary endpoint and overall design. The rest of the authors made substantial contributions to the study design and manuscript preparation, as detailed in the “Declarations” section of the manuscript. The scientific committee is also in charge of trial oversight, with AR-M coordinating day-to-day issues. A professional monitor, belonging to the research support office of the Hospital acting as study sponsor, will monitor a subset of all data collected, as described in the study protocol.

## Introduction

The debut of telemedicine into routine practice stretches back over three decades [1]. However, the use of this modality of care delivery has exponentially grown in the past few years, accompanied by a remarkable body of literature reporting the benefits of different types of telemedicine in the management and follow-up of various chronic and acute conditions [2–6].

The concept of telemedicine encompasses multiple information and communication technologies (ICT) solutions for monitoring patients remotely [7]. Among them, video conferencing and phone calls (commonly regarded as video consultation and teleconsultation, respectively) are candidates to re-shape care pathways in the mid-term, thus mitigating the expected overwhelming of healthcare systems associated with the upcoming demographic shift [8, 9]. In the past 2 years, the public health emergency derived from the COVID-19 pandemic has boosted the implementation of remote consultation systems to avoid crowding of healthcare centers [10–12], thus accelerating the entrance of this modality of care delivery in routine practice.

The rapid implementation of remote consultations in many healthcare systems of high-income countries has not been accompanied by high-quality evidence regarding its effectiveness and safety. Thus, although thousands of studies have described the benefits and usage of various types of remote consultation, very few correspond to randomized-controlled designs, and the assessment of potential harms of remote consultation has been typically listed as secondary—often descriptive—outcomes [13, 14]. We present herein a study protocol of a randomized controlled trial to assess the safety of remote consultations (both video and teleconsultation) in the follow-up of patients in the hospital setting.

## Design

### Study setting and participants

The ECASeT trial (*Ensayo Clínico abierto y Aleatorizado para valorar la Seguridad de la Teleconsulta, frente a la consulta clínica presencial* in Spanish) is aimed at testing safety of remote consultations, based on its non-inferiority compared with face-to-face consultation. The ECASeT trial will be conducted in four centers in the healthcare area of *Alt Penedès-Garraf* in Catalonia (North-East Spain): three secondary hospitals (Hospital Sant Antoni Abat, Hospital Sant Camil, and Hospital Comarcal de l’Alt Penedès) and a rehabilitation center, all part of Healthcare Consortium Alt Penedès Garraf (CSAPG). All study sites belong to the public network of hospitals and primary care centers of the Catalan Health Service and provide universal healthcare to a catchment population of 247,357 inhabitants. Hospital departments from all areas (i.e., locomotive, internal medicine, surgery, gynecology, and pediatrics) are involved in the study.

Participants will be screened from all individuals, irrespective of age and sex, who require follow-up in outpatient consultations of any of the departments involved in the study. Eligibility criteria have been established based on the local guidelines for screening patients for remote consultation [15]. Briefly, to be eligible for remote consultation, patients have to have adequate digital literacy at the physician discretion and technological capacity to use the video conferencing software and undergo a type of follow-up that, due to the moderate complexity of the pathology, does not require physical examinations. Patients followed up in more than three departments, those with visual or hearing impairments that may hamper patient-physician communication, and those enrolled in another clinical trial that requires an experimental intervention during the follow-up will be excluded from the study.

### Interventions

Participants will be randomly allocated into one of the two study arms: control (i.e., face-to-face consultation) and remote consultation (including teleconsultation, and video consultation). All patients, irrespective of the consultation type, will receive short text message reminders a few days before the visit.

Participants allocated into the control arm will be scheduled with face-to-face appointments as usual. All phone calls (except reminders for scheduled visits) performed by the physician during the study will be noted in the electronic case report form (eCRF). Per protocol, participants allocated in the control arm can receive up to 25% of physician-doctor interactions by phone (that is, one phone call allowed for three face-to-face appointments).

Participants allocated in the remote consultation arm will be scheduled remote visits, either phone or video consultation. In case of phone visits, the physician will call the main phone number provided by the patient and, in case of not receiving an answer, other phone numbers listed as contact numbers. In case of video consultation, the participant will receive an additional text message with the visit link. When clicking the link, a website with the video consultation system embedded will open in the default browser of the user. The video consultation platform of the physician pops up a message indicating that the patient is in the virtual waiting room. Patients will remain in the virtual waiting room until the physician starts the visit. A technical assistance is readily available for physicians who experience troubles with the system. In case the patient does not show up to the video consultation after a reasonable time, the physician calls the patient by phone. The date and hour of the phone call and the reason for the inability to connect are recorded in the eCRF.

Participants allocated in the remote consultation arm that require face-to-face visits will be excluded from the per-protocol analysis, and the reason for the face-to-face visit will be recorded in the eCRF. Participants allocated in the remote consultation arm and scheduled with a phone visit cannot be visited by video, whereas those scheduled for a video consultation can undergo up to 25% of phone visits. Phone contacts and their reason in patients in the video consultation arm will be recorded in the eCRF.

Non-scheduled visits will be defined as any contact between the patient and the physician that occurs before the date established in the previous follow-up visit. Non-scheduled visits will be performed according to the study arm and type of remote consultation (i.e., phone or video) in which the patient has been allocated and will be considered for the analysis.

### Screening, allocation, and blinding

All consecutive patients who visited any of the participating departments between May 2022 and May 2024 will be informed about the study design and objective and will be offered to enroll in the trial. Patients who agree to participate will provide their informed consent, which will be signed in case of a face-to-face screening visit and oral in case of a remote visit. Patients who reject enrollment will be recorded in an anonymous registry. Once signed the informed consent, the investigator will assess the eligibility criteria and record them in the eCRF.

Patients will be randomly allocated in either of the study arms irrespective of the modality of the screening visit. Randomization will be balanced by medical specialty to prevent biases towards a particular diagnosis within study arms. To ensure a minimum number of participants who visited through video consultation, participants allocated to the remote consultation arm will be subsequently randomized to either video consultation or remote modality selection at physician discretion at a 3:7 ratio.

Centralized randomization will be automatically conducted by the randomization module of the REDCAP system used for the eCRF. The allocation will be informed to the administrative service, which will schedule all visits indicated by the physician during the investigated period following the criteria of each study arm. The health condition for which a patient has attended the screening visit will be considered the baseline disease for the study purposes.

Although study participants will be informed about the overall study objectives in the patient information sheet, they will not be aware of the study endpoints. In addition, the statisticians performing the analysis will be blinded to participant allocation. No rules for unblinding have been pre-defined in the study protocol.

### Data sources

To reduce the workload of participating investigators during visits, variables recorded in the eCRF will be limited to those that cannot be accurately retrieved from electronic health records (EHR), which will include both local hospital records and the dataset of the Catalan Health Surveillance System (CHSS), a central database of the Catalan Health System. By the time of submitting the study protocol to the ethics committee, all four study sites have separate and different EHRs. Throughout the following year, all the systems are to be unified into a single one and all the data from the different systems will be migrated. These EHR collect all the data related to the care received in the centers, clinical course, prescription, diagnostic images, laboratory tests, and administration, among others. The CHSS systematically collects and stores healthcare data on health status and resource utilization from all healthcare providers. The CHSS was originally designed for healthcare planning and has an automated validation system to identify internal inconsistencies between variables. Since the CHSS registries are used for control of capitation payments, they undergo external audits to ensure provider payment accuracy [16].

### Outcomes and variables

Table 1 summarizes the definitions and data sources of primary and secondary outcomes assessed in this trial. Complications and adverse reactions will be considered only if related to the baseline disease (i.e., the health condition for which the first study visit was scheduled). To prevent interference with routine practice, the investigator will have the option of recording the reference disease in a free-text field in the eCRF. The option of completing a field in English or Spanish linked to the ontologies of the ICD10 will also be available. If this field is not filled in, the ICD-10 code will be extracted from the free-text field. The investigator will also record the stage (if applicable) of the baseline disease and its severity.

**Table 1:** Study outcomes and data sources

**Item**	**Domain definition**	**Measure and metrics**	**Data sources**^**a**^
**Primary outcome**			
Rate of complications^b^ of the baseline disease	A secondary disease, an accident, or a negative reaction (including adverse reactions to treatment) occurring during the course of the baseline disease and usually aggravating the illness.	Measure: events, as recorded in the eCRF. Metrics and aggregation: event rate (i.e., proportion of patients experiencing an event within each arm and the follow-up period).	Investigator-reported in the eCRF Local EHR.
**Secondary outcomes**			
Rate of severe complications of the baseline illness	A complication with significant impact on patient’s life and/or that require intervention by a healthcare professional	Measure: events, as recorded in the eCRF. Metrics and aggregation: event rate (i.e., proportion of patients experiencing an event within each arm and the follow-up period).	Investigator-reported in the eCRF Local EHR.
Rate of severe adverse reactions related to the treatment of the baseline condition	An undesired health event related to a drug administered to treat the baseline health condition	Measure: events, as recorded in the eCRF. Metrics and aggregation: event rate (i.e., proportion of patients experiencing an event within each arm and the follow-up period).	Investigator-reported in the eCRF
Rate of preventable hospitalizations	Hospitalizations due to illnesses secondary to the chronic diseases that can be managed in the outpatient setting^c^	Measure: events, as recorded in the eCRF. Metrics and aggregation: event rate (i.e., proportion of patients experiencing an event within each arm and the follow-up period).	Local EHR
Non-scheduled medical encounters related to poor control of the baseline disease	The following events will be considered: • Non-scheduled appointments (phone, videoconference, or face-to-face). • Visits to primary care services. • Visits to the emergency room. • Hospital admissions.	Measure: events, as recorded in the eCRF. Metrics and aggregation: event rate (i.e., proportion of patients experiencing an event within each arm and the follow-up period).	Investigator-reported in the eCRF Local EHR
Pharmaceutical prescriptions	The number of new prescriptions and renewals will be collected, along with the type of pharmaceutical prescription: long-term, acute process, or pro re nata.	Measure: ATC groups prescribed Metrics and aggregation: difference in number of ATC groups between baseline and last visit; the outcome will be measured as the mean and standard deviation, and median and interquartile range (i.e., 25^th^ and 75^th^ percentiles).	Administrative records.
Usability	Usability parameters will be collected using two approaches: • Computer System Usability Questionnaire. • Technical incidents reported by the investigators.	Measure: Questionnaire score and number of technical incidents. Metrics and aggregation: score, expressed as the mean and standard deviation, and median and interquartile range (i.e., 25^th^ and 75^th^ percentiles). Cumulative number of incidents.	Phone call administration by trained interviewers to 10% of the study sample. Investigator report to the eCRF.
Satisfaction	Satisfaction survey CSAPG included in the satisfaction survey plan of the Catalan Health System.	Measure: Questionnaire score. Metrics and aggregation: score, expressed as the mean and standard deviation, and median and interquartile range (i.e., 25th and 75th percentiles).	Phone call administration by trained interviewers to 10% of the study sample.
Care provision expenditure	Expenses related to each of the interventions.	Measure: cost in euros of the year. Metrics and aggregation: mean and standard deviation per patient.	Administrative records.

*EHR* electronic health record

^a^In outcomes gathered from more than one data source, discrepancies or inconsistencies between data sources will be addressed by the scientific committee before the closure of the database

^b^The severity of complications and adverse events will be recorded using a scale with four categories: (a) MILD: does not limit activity and does not require medical follow-up; (b) MODERATE: mild-to-moderate impact on activity or requires minimal medical intervention or monitoring; (c) SEVERE: significant impact on activity or requires medical care; (d) VERY SEVERE: life-threatening or results in major disability or significant incapacity for the subject

^c^The list of conditions considered has been retrieved from ambulatory care sensitive conditions and include ICD-10 categories related to ischemic heart (I20, I240, I248, I249), asthma (J45, J46), COPD (J20, J41, J42, J43, J44, J47), heart failure (I110, I50, J81), seizures (G40, G41, R56, O15), diabetes (E100-E108, E110-E118, E120-E128, E130-E138, E140-E148), hypertension (I10, I119), and iron-deficiency anemia (D501, D508, D509)

The follow-up period planned by the investigator for the baseline disease will be retrieved from the local EHR. Other variables collected from EHRs at baseline will include sex, age, area of residence, the number of active prescriptions at the end of the screening visit, and the comorbidity burden, summarized using the Adjusted Morbidity Groups (AMG). Briefly, the AMG is a case-mix tool that generates a summary index of health risk by considering all chronic conditions and recent acute diagnostic codes [17]. The tool allows stratifying patients into mutually exclusive health-risk groups and has shown a high predictive capacity of healthcare and resource use outcomes, including mortality, hospitalization, visits to primary care, polypharmacy, and overall expenditure [18, 19].

For homogeneity regarding the rate of preventable hospitalizations, we considered all hospitalizations due to any of the ICD-10 diagnostic codes listed as ambulatory care sensitive conditions [20] managed in the participating departments, including conditions related to ischemic heart disease (I20, I240, I248, I249), asthma (J45, J46), chronic obstructive pulmonary disease (COPD) (J20, J41, J42, J43, J44, J47), heart failure (I110, I50, J81), seizures (G40, G41, R56, O15), diabetes (E100-E108, E110-E118, E120-E128, E130-E138, E140-E148), high blood pressure (I10, I119), and iron-deficiency anemia (D501, D508, D509).

Usability will be assessed by passive and active surveillance. Study investigators will record non-attendances, as well as all technical incidences that occurred during the visit in an unstructured field of the eCRF. For active surveillance, a trained interview panel will contact by phone a subset of 100 participants randomly selected from all patients who have completed the final visit. Interviewers will administer the computer system usability questionnaire (CSUQ), a validated questionnaire aimed at measuring the users’ satisfaction with the usability of computer systems in scenario-based usability studies [21].

The users’ satisfaction with the quality of care received will be assessed using a validated tool encompassed within the satisfaction survey plan of the Catalan Health Service [22]. The questionnaire consists of 32 items that combine binary and multiple choice questions, 10-point assessment scales, and 2 open questions. An interview panel will administer the questionnaire by phone to a subset of 300 participants (100 per arm), randomly selected from all participants who have completed the final visit.

### Participant timeline

**Fig. 1:**
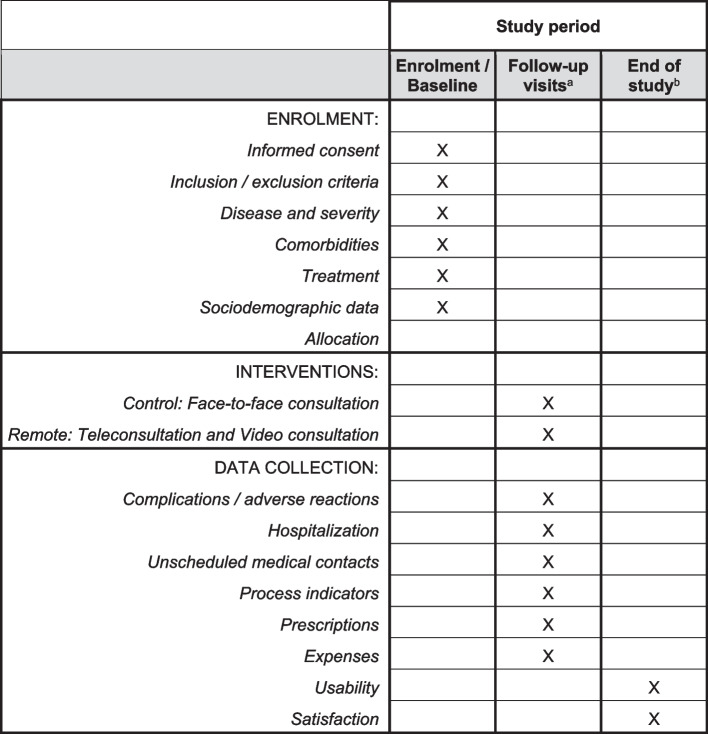
Participant timeline ^a^The frequency of the follow-up visits will be determined by the physician based on the diagnostic and therapy of the baseline disease and will receive a reminder (either SMS or phone call) before each visit. ^b^Either the follow-up visit closest to 12 ± 2 months after enrollment or before in case of discharge or early discontinuation

Figure 1 summarizes the participant timeline. All participants, irrespective of the study arm, will be scheduled follow-up visits at a frequency determined by the physician based on the diagnostic and therapy of the baseline disease and will receive a reminder (either SMS or phone call) before each visit. Physicians can schedule and cancel appointments at their discretion. At least three visits should be scheduled in the study setting: the baseline visit (i.e., screening visit), intermediate visits (pre-defined by the physician based on the follow-up needed for managing the baseline condition), and the final visit (i.e., either the follow-up visit closest to 12 ± 2 months after enrollment or before in case of discharge or early discontinuation).

In addition to voluntary withdrawal, the following situations will lead participants in the remote consultation arms to discontinue the study: a change in their clinical status that requires face-to-face consultation and not following the procedures for remote consultation adequately. Patients allocated in the face-to-face arm will discontinue in case their health status discourages moving to the hospital for follow-up visits. The reasons for ending the study will be recorded in the eCRF.

The study will be temporarily interrupted if the local authorities or healthcare center managers decide to ban or limit face-to-face consultations because of the rapid spread of an infectious agent. This interruption can be partial in case banning face-to-face consultations does not affect all hospital services. The study will resume when face-to-face consultations are restored, and there is enough healthcare activity for adequate recruitment in all study arms. In case of a temporal interruption, the study will be automatically extended for a time that is at least equivalent to the interruption time.

The independent ethics committee can interrupt the study at any time in the advent of risk for participants, and the study sponsor can do it as well because of financial issues.

### Data management and analysis

All data recorded in the eCRF will be stored in a REDCap platform hosted in a local server. The REDCap data will be transferred to a database, which will also contain the data extracted from the local EHR and the CHSS registries. All registries will be linked through the identification number of the Catalan Health Service.

Data recorded in the eCRF will be monitored for completeness and quality by a professional monitor working at the research office of the Hospital acting as sponsor. We will schedule periodic visits to assess the adherence of investigators to the study protocol and good clinical practices. The clinical monitor will release reports at the end of each visit.

A statistical analysis plan, with detailed analyses for each study outcome, will be performed after the last patient has attended the final visit and before the closure of the database.

The primary objective will be assessed using a non-inferiority analysis of the cumulative incidence of complications of the baseline disease between remote consultation (video and teleconsultation arms) and face-to-face consultation, using a non-inferiority margin of 2%. The analysis will be based on a modified binomial test to assess the non-inferiority of an experimental intervention vs. a control group in a three-arm trial [23]. The primary analysis will be conducted on a per-protocol study sample, which will include all participants who have finished the study and have not been withdrawn because of non-allowed visits using modalities other than scheduled. The intention-to-treat population, used for sensitivity analyses, will consist of all randomized patients completing at least one visit. Since the analysis will be based on an event rate, missing data will not be imputed.

The primary analysis will be replicated with patients stratified according to the following characteristics: hospital department, diagnostic, disease duration (chronic vs. acute or sub-acute), anatomic-therapeutic treatment groups, and GMA health risk group. The clinical and demographic characteristics of study participants will be described and compared between excluded/withdrawn participants.

The statisticians will be blinded for the allocation arm. The type I error will be set at 2.5% for the primary analysis and 5% for the rest of the analyses. All analyses will be conducted using R software (version 4.0 or higher) [24]. Data collected during the ECASeT trial will not be re-used.

### Sample size considerations

The sample size was estimated based on the primary endpoint of cumulative incidence of complications related to the baseline disease. The sample size analysis was computed with R software [24], following the approach described by Steen A. Julious for non-inferiority trials [25]. Although there is little available information regarding this outcome in the general population of chronic patients, we expect 10% of events in face-to-face consultations [26–28] and 12% in the remote consultation arm (based on routine practice of the research team). Assuming a type-I error of up to 2.5% and a statistical power of 80%, 1068 subjects per arm should be analyzed to reject the non-inferiority hypothesis with a non-inferiority margin of 2% and assuming 10% of participants lost to follow-up.

Altogether, the participating centers schedule 252,000 routine follow-up visits during a period equivalent to the study, corresponding to 121,400 different patients. Therefore, the recruitment of the estimated 2136 patients is considered within the capabilities of the participating centers.

Owing to the scarcity and heterogeneity of available data about the primary endpoint, a blinded interim analysis will be conducted when 50% of the total sample has been recruited to revise the assumptions used to calculate the sample size, as proposed in the E9 Guide of the ICH on Statistical Principles for Clinical Trials [29].

### Ethical study conduct

We will collect only the data necessary to achieve the study objectives. All data will be handled according to the Spanish Organic Low 03/2018 for the protection of personal data and assurance of digital rights (LOPD-GDD) and the General Data Protection Regulation 2016/679 on data protection and privacy for all individuals within the European Union and the local regulatory framework regarding data protection. No situations classified at high risk of violation of the rights and freedoms of patients according to the LOPD-GDD are foreseen. Nevertheless, considering that the study will include video conferencing, we will perform the risk analysis report requested by the LOPD-GDD. The data generated during the study will be stored for 5 years after the study ends unless the Independent Ethics Committee or the local regulatory framework establishes otherwise.

The study protocol has been approved by the Independent Ethics Committee of the Bellvitge Hospital (L’Hospitalet de Llobregat, Spain) (ref. PR215/21). All changes made to the study design after protocol approval will be notified to the Independent Ethics Committee and documented as an amendment in the ClinicalTrials.gov registry. The scientific committee and the sponsor are responsible for communicating all amendments that have been approved by the Independent Ethics Committee to the rest of the investigators, as well as, patients already enrolled in the trial if deemed necessary.

When offered to enter the study, all participants will be provided with a patient information sheet (Additional file 2). All patients who accept to participate in the study will provide informed consent before entering the study. In case the first visit is remote, consent will be oral, otherwise, written. Participants younger than 18 years will sign an informed assent, and their legal guardian will provide the corresponding consent.

The members of the scientific committee commit to public dissemination of the trial results. Therefore, upon trial completion and analysis, a report will be submitted in a peer-reviewed, open-access journal, regardless of the trial results. The scientific committee will be responsible for making decisions regarding authorship, which will be in agreement with the authorship criteria of the International Committee of Medical Journal Editors. The report will also be sent to local authorities responsible for healthcare planning.

## Discussion

In this article, we describe the key elements of the design of an RCT to assess the safety of two modalities of synchronous remote consultations: video consultation and teleconsultation. Trials investigating the benefits and—less frequently—risks of remote consultation typically focused on a specific clinical condition [30–32]. Alternatively, we seek to address whether remote consultations can be considered overall safe in clinical conditions commonly followed up in a secondary hospital. While broadening the scope of the research, this approach raises notable challenges regarding the study design that are worth being discussed.

One of these challenges is the selection of the appropriate study outcome. Unlike the assessment of safety in drug therapy, commonly based on the number of adverse events related to the given drug, care delivery through remote consultation modalities lacks specific pre-defined harms that can be monitored. In the context of a particular diagnostic, the safety of a follow-up could be understood as the capacity of the care pathway to prevent an unexpected worsening of the disease (e.g., asthma exacerbations or cardiac arrest events). However, in our study, which encompasses many chronic conditions, the definition of the safety outcome had to include any possible complication that can be attributed to inadequate control of the baseline condition. We considered that listing all potential complications associated with the baseline conditions allowed in the trial would hamper excessively the study conduct and constrain the repertoire of possible complications. Instead, we chose a broad definition of complication [33]. To prevent unnoticed complications, we will complement the investigator’s assessment with a systematic screening of all events recorded in the EHR within the follow-up period, which are likely to include complications not recorded in the eCRF, such as visits to the emergency room, and primary care services related to the baseline condition.

The definition of a candidate profile that is suited for remote consultation has been identified among the important challenges of remote consultation deployment [34]. In the research setting, this issue results in difficulty in defining the target population and, therefore, the selection criteria for recruitment. In response to the high demand for remote consultations in our area during the COVID-19 pandemic, the Catalan Health System developed a set of guidelines for helping clinicians and managers of healthcare centers implementing and offering video and teleconsultation appropriately [15, 35]. These guidelines, agreed by different stakeholders, recommend offering remote consultation only to patients without severe or unstable diseases. While these criteria exclude patients at higher risk of experiencing complications, they are unlikely to be offered remote consultation in the real world. Therefore, including them might result in a biased view of the safety of remote consultation in routine practice. Nevertheless, the applicability of the results obtained from our study shall be constrained to candidates to be followed up by remote consultation according to the local guidelines. Briefly, these guidelines identify as candidates all patients without severe diseases who are predisposed to contact the physician remotely.

Aside from the little precedent for making decisions on trial design, our study is challenged by the huge sample to be enrolled for achieving enough statistical power for a primary outcome that is expected to occur in a low frequency. Although the assumptions for sample size calculation will be revised in an interim analysis, as proposed by ICH E9 guidelines [29], recruiting such a huge number of patients is among the main risks of trial success. The risk of not reaching a sample size high enough for a powered analysis might be worsened by other aspects aside from recruitment, including a lower frequency of the primary outcome than expected, short follow-up periods established by the clinician, and high frequency of arm switch because of care needs.

In summary, the study protocol of this RCT, which is novel in assessing the safety of remote consultation as a primary endpoint in general consultations and with powered sample size, provides design insights that will hopefully aid researchers in making decisions in future trials for assessing remote consultation.

## Trial status

By the time of submitting the manuscript, the trial is recruiting patients. No interim analyses have been done yet.

Current protocol version: 2.0 (07/02/2023).

Recruitment started on November 01, 2021, and was interrupted until the protocol amendment on February 07, 2023.

Expected date for ending recruitment: February 28, 2025.

### Supplementary Information

**Additional file 1.** WHO dataset ECASeT.

**Additional file 2.** Information sheet.

**Additional file 3.** SPIRIT Checklist.

## Data Availability

The study protocol submitted to the Ethics Committee is available from the corresponding author upon request.

## Abbreviations

AMG: Adjusted Morbidity Groups
CHSS: Catalan Health Surveillance System
CSUQ: Computer system usability questionnaire
COVID-19: Coronavirus disease
ECASeT: *Ensayo Clínico abierto y Aleatorizado para valorar la Seguridad de la Teleconsulta, frente a la consulta clínica presencial*
eCRF: Electronic case report form
EHR: Electronic health records
ICD: International Classification of Diseases
ICT: Information and communication technologies

## References

[1] Nesbitt TS, Katz-Bell J. History of Telehealth. In: Rheuban K, Krupinski EA. eds. Understanding Telehealth. McGraw Hill; https://accessmedicine.mhmedical.com/content.aspx?bookid=2217§ionid=187794434. Accessed 9 Oct 2023.

[2] Brebner JA, Brebner EM, Ruddick-Bracken H. Accident and emergency teleconsultation for primary care - a systematic review of technical feasibility, clinical effectiveness, cost effectiveness and level of local management. J Telemed Telecare. 2006;12. Epub ahead of print. 10.1258/135763306777978542. 10.1258/135763306777978542 16884562

[3] Verhoeven F, Van Gemert-Pijnen L, Dijkstra K, et al. The contribution of teleconsultation and videoconferencing to diabetes care: a systematic literature review. J Med Internet Res. 2007;9. Epub ahead of print. 10.2196/jmir.9.5.e37. 10.2196/jmir.9.5.e37 PMC2270420 18093904

[4] Verhoeven F, Tanja-Dijkstra K, Nijland N (2010). Asynchronous and synchronous teleconsultation for diabetes care: a systematic literature review. J Diabetes Sci Technol.

[5] Melian C, Kieser D, Frampton C, et al. Teleconsultation in orthopaedic surgery: a systematic review and meta-analysis of patient and physician experiences. J Telemed Telecare. 2020. Epub ahead of print. 10.1177/1357633X20950995. 10.1177/1357633X20950995 32873138

[6] Gupta T, Gkiousias V, Bhutta MF (2021). A systematic review of outcomes of remote consultation in ENT. Clin Otolaryngol.

[7] NEJM Catalyst. What is Telehealth? Innovations in Care Delivery. 2018. https://catalyst.nejm.org/doi/full/10.1056/CAT.18.0268 (Accessed 23 Sept 2021).

[8] Shaw S, Wherton J, Vijayaraghavan S (2018). Advantages and limitations of virtual online consultations in a NHS acute trust: the VOCAL mixed-methods study. Heal Serv Deliv Res.

[9] Levy S, Bradley DA, Morison MJ (2002). Future patient care: tele-empowerment. J Telemed Telecare.

[10] Bitar H, Alismail S (2021). The role of eHealth, telehealth, and telemedicine for chronic disease patients during COVID-19 pandemic: a rapid systematic review. Digit Heal.

[11] Hong Z, Li N, Li D (2020). Telemedicine during the COVID-19 pandemic: experiences from Western China. J Med Internet Res.

[12] Sust PP, Solans O, Fajardo JC (2020). Turning the crisis into an opportunity: digital health strategies deployed during the COVID-19 outbreak. JMIR Public Heal Surveill.

[13] Totten AM, Womack DM, Eden KB, et al. Telehealth: mapping the evidence for patient outcomes from systematic reviews. Technical Briefs, No. 26. Rockville: Agency for Healthcare Research and Quality (US); 2016. 27536752

[14] McLean S, Sheikh A, Cresswell K (2013). The impact of telehealthcare on the quality and safety of care: a systematic overview. PLoS ONE.

[15] Piera-Jiménez J, Berdun J, Solans O, et al. Facilitating the implementation of remote consultations: development of evidence-informed visual guidelines. Proceedings of the 4th UK Implementation Science Research Conference. Impl Sci. 2016;16(104). 10.1186/s13012-021-01163-7.

[16] Vela E. Stratification and morbidity database. AQuAS Blog (Agency for Health Quality and Assessement of Catalonia). 2016. https://blog.aquas.cat/2016/03/31/morbidity-database-2/?lang=en.

[17] Monterde D, Vela E, Clèries M (2016). Adjusted morbidity groups: a new multiple morbidity measurement of use in primary care. Aten Primaria.

[18] Monterde D, Vela E, Clèries M (2020). Multimorbidity as a predictor of health service utilization in primary care: a registry-based study of the Catalan population. BMC Fam Pract.

[19] Vela E, Clèries M, Monterde D (2021). Performance of quantitative measures of multimorbidity: a population-based retrospective analysis. BMC Public Health.

[20] Purdy S, Griffin T, Salisbury C (2009). Ambulatory care sensitive conditions: terminology and disease coding need to be more specific to aid policy makers and clinicians. Public Health.

[21] Lewis JR (1995). Computer system usability questionnaire. Int J Hum Comput Interact.

[22] CatSalut. Servei Català de la la Salut. Enquestes de satisfacció. Catsalut. Servei Català de la Salut. CatSalut. Barcelona: Servei Català de la Salut; 2018. https://catsalut.gencat.cat/ca/coneix-catsalut/presentacio/instruments-relacio/valoracio-serveis-atencio-salut/enquestes-satisfaccio/index.html#googtrans(ca%7Cen). Accessed 3 May 2021.

[23] Kieser M, Friede T (2007). Planning and analysis of three-arm non-inferiority trials with binary endpoints. Stat Med.

[24] R Core Team. R: a language and environment for statistical computing. R Foundation for Statistical Computing: Vienna. 2021. https://www.r-project.org (Accessed 20 Dec 2021).

[25] Julious SA (2009). Sample size for clinical trial. Sample size for clinical trial.

[26] Gandhi TK, Weingart SN, Borus J (2003). Adverse drug events in ambulatory care. N Engl J Med.

[27] Hanlon JT, Schmader KE, Koronkowski MJ (1997). Adverse drug events in high risk older outpatients. J Am Geriatr Soc.

[28] Hutchinson TA, Flegel KM, Kramer MS (1986). Frequency, severity and risk factors for adverse drug reactions in adult out-patients: a prospective study. J Chronic Dis.

[29] International Conference on Harmonization. ICH E9 statistical principles for clinical trials. https://www.gmp-compliance.org/files/guidemgr/E9_Guideline.pdf (Accessed 21 Jan 2022).

[30] Boman K, Olofsson M, Berggren P (2014). Robot-assisted remote echocardiographic examination and teleconsultation: a randomized comparison of time to diagnosis with standard of care referral approach. JACC Cardiovasc Imaging.

[31] Fatehi F, Gray LC, Russell AW (2015). Validity study of video teleconsultation for the management of diabetes: a pilot randomized controlled trial. Diabetes Technol Ther.

[32] Dichmann Sorknaes A. The effect of tele-consultation between a hospital-based nurse and a COPD patient. In: Studies in Health Technology and Informatics. Stud Health Technol Inform; 2016, p. 883–884. 27332391

[33] The American Heritage® Medical Dictionary. Complications. 2007. https://medical-dictionary.thefreedictionary.com/Complications (Accessed 17 Sept 2021).

[34] Greenhalgh T, Rosen R, Shaw SE (2021). Planning and evaluating remote consultation services: a new conceptual framework incorporating complexity and practical ethics. Front Digit Heal.

[35] Berdún J. Recommendations for the use of non-face-to-face care channels. TIC Salut Social. 2020. https://ticsalutsocial.cat/en/noticia/recommendations-for-the-use-of-non-face-to-face-care-channels/ (Accessed 21 Sept 2021).

