# Supplementary material for: Study protocol of a randomized controlled trial to assess safety of teleconsultation compared with face-to-face consultation: the ECASeT study
Source: Trials. 2023 Dec 8;24:797. doi: 10.1186/s13063-023-07679-1 (PMC10704815; doi:10.1186/s13063-023-07679-1)
Supplement: Supplementary file 1 — Additional file 1. WHO dataset ECASeT. [file 13063_2023_7679_MOESM1_ESM.pdf]

WHO Dataset

**Primary Registry and Trial Identifying Number**	
Name of Primary Registry	ClinicalTrials.gov
Primary Register ID	NCT05094180
**Date of Registration in Primary Registry**	2021-10-26
**Secondary Identifying Numbers**	
Identifiers assigned by the sponsor	CSAPG-15
Other identifiers	ECASeT
**Source(s) of Monetary or Material Support**	Consorci Sanitari de l'Alt Penedès i Garraf
**Primary Sponsor**	Consorci Sanitari de l'Alt Penedès i Garraf
**Secondary Sponsor(s)**	None.<br><br>Collaborator: Institut Català de la Salut
**Contact for Public Queries**	Alejandro Rodríguez-Molinero, PhD<br>Phone Number:938960025 ext 47158<br>Email Address: arodriguez@csapg.cat
**Contact for Scientific Queries**	Alejandro Rodríguez-Molinero, PhD<br>Consorci Sanitari de l'Alt Penedès i Garraf<br>Phone Number:938960025 ext 47158<br>Email Address: arodriguez@csapg.cat
**Public Title**	Open Label Randomized Clinical Trial to Assess the Safety of Teleconsultation (ECASeT)
**Scientific Title**	Open Label Randomized Clinical Trial to Assess the Safety of Teleconsultation, Compared to the Traditional Clinical Consultation
**Countries of Recruitment**	Spain
**Health Condition(s) or Problem(s) Studied**	Various Clinical Conditions Commonly Followed up in a Secondary Hospital
**Intervention(s)**	Two study arms:<br><br>• **Face-to-face appointment**: Participants will be scheduled with face-to-face appointments as usual (control arm). All phone calls (except reminders for scheduled visits) performed by the physician during the study will be recorded.<br><br>• **Remote consultation,** either (tele or video consultation): Participants allocated in the remote consultation arm will be scheduled phone call or video consultation visits.<br><br>- Phone visits will be scheduled in a date and time slot in which the physician will contact; the physician will call the main phone number provided by the patient

	and, in case of not receiving an answer, other phone numbers listed as contact numbers.<br>- To conduct video consultations, patients will be provided with an app, which can be installed in mobile phone, for safe videoconference. At the moment of the videoconsultation, the patient will be provided with a link to the meeting through the video conference app. Patients will remain in the virtual waiting room until the physician starts the visit.
**Key Inclusion and Exclusion Criteria**	Key Inclusion Criteria:<br><br>● Men and women of any age.<br>● Possibility of making consultations by telephone, at the discretion of the investigator, taking into account the severity and complexity of the baseline pathology and the objective of health care.<br>● Forecast to carry out follow-up in external consultation.<br>● Agreement to be attended through a non-face-to-face system.<br>● Ability to connect to the video consultation system.<br>● Device compatible with the video consultation system.<br>● Possibility of collaborating in the necessary evaluations.<br>● Legal capacity to give informed consent.<br>● Signature of the informed consent for inclusion of the study.<br><br>Exclusion Criteria:<br><br>● Need to carry out physical examinations, visualize the patient in person or perform techniques that involve face-to-face visits.<br>● Need for face-to-face consultation due patient's clinical situation.<br>● Follow-up by more than three medical specialists.<br>● Visual, hearing or functional impairments that may hamper patient-physician communication.<br>● Patients enrolled in another clinical trial that requires an experimental intervention during the follow-up.
**Study Type**	● Type of study: interventional<br>● Study design:<br>o Randomized

	○ Unmasked<br>○ Parallel assignment<br>○ Purpose<br>● Phase (if applicable)<br><br>The allocation concealment mechanism and sequence generation will be documented.
**Date of First Enrollment**	2021-11-01
**Sample Size**	● Number of participants that the trial plans to enrol in total: 2136<br>● Number of participants that the trial has enrolled: 483
**Recruitment Status**	● Recruiting
**Primary Outcome(s)**	● Complications of the underlying disease [Time Frame: 12 months]<br>○ Frequency of complications of the underlying disease, including adverse reactions to the treatment of the underlying disease
**Key Secondary Outcomes**	● Severe complications of the baseline disease [Time Frame: 12 months]<br>○ Frequency of severe complications of the underlying disease.<br>● Treatment Serious Adverse Event [Time Frame: 12 months]<br>○ Frequency of Serious Adverse Events related to the underlying disease's treatment<br>● Avoidable hospitalizations [Time Frame: 12 months]<br>○ Total and avoidable hospitalizations, secondary to ambulatory care sensitive conditions<br>● Number of unscheduled medical contacts [Time Frame: 12 months]<br>○ Count of unscheduled medical contacts (phone calls, unscheduled visits, ER visits, and admissions) registered in the hospital information system<br>● Care process indicators [Time Frame: 12 months]<br>○ Number of consultations made in a format different from the one initially foreseen (face-to-face, telephone consultation or video consultation) Number of empty consultations (the patient does not attend) Number of patients who interrupt their outpatient follow-up Number of prescriptions and type of prescription

	• Healthcare expenditure [Time Frame: 12 months]<br>○ Estimated healthcare expenditure associated with each of the three interventions<br>• Usability of video consultation system [Time Frame: 12 months]<br>○ Usability of video consultation system, measured by using the Computer System Usability Questionnaire<br>• Satisfaction of users (health professionals and patients) with the process of clinical care [Time Frame: 12 months]<br>○ Degree of satisfaction of users (health professionals and patients) with the process of clinical care, measured by using the satisfaction questionary of the Pla d'enquestes de satisfacció del CatSalut
**Ethics Review**	The ethics review process information of the trial record in the primary register database. It consists of:<br><br>○ Status: Approved<br>○ Date of approval: 10 JUN 2021<br>○ Name and contact details of Ethics committee(s):<br><br>Comité de ética de la investigación (CEI) con medicamentos (CEIm) del Hospital Universitario de Bellvitge.<br><br>Contact: presidenciaceic@bellvitgehospital.cat
**Completion date**	2023-10-30 (Foreseen final data collection date for primary outcome measure)
**Summary Results**	Not applicable
**Individual patient data (IPD) sharing statement**	
Plan to share IPD (Yes, No)	Yes
Plan description	IPD will be shared to verify the quality of the work, but not for future research.
