# Supplementary material for: Study protocol of a randomized controlled trial to assess safety of teleconsultation compared with face-to-face consultation: the ECASeT study
Source: Trials. 2023 Dec 8;24:797. doi: 10.1186/s13063-023-07679-1 (PMC10704815; doi:10.1186/s13063-023-07679-1)
Supplement: Supplementary file 2 — Additional file 2. Information sheet. [file 13063_2023_7679_MOESM2_ESM.pdf]

Participant Information Sheet

STUDY TITLE	Study Protocol of a Randomized Controlled Trial to Assess Safety of Teleconsultation Compared with Face-to-Face Consultation. The ECASeT Study
STUDY CODE	CSAPG-15
SPONSOR	Consorci Sanitari de l'Alt Penedès i Garraf
INVESTIGATOR	
CENTER	
CONTACT	

We would like to provide you with information about a research study in which you are invited to participate. The study has been approved by a Research Ethics Committee, in accordance with current legislation.

Our intention is to provide you with the correct and sufficient information so that you can decide whether to accept or decline participation in this study. Please read this information sheet carefully, and we will address any questions that may arise. Additionally, you may consult with whomever you deem appropriate.

Voluntary Participation

You should know that your participation in this study is voluntary, and you can choose not to participate. If you decide to participate, you can change your decision and withdraw your consent at any time without affecting your relationship with your doctor or your healthcare.

We invite you to participate because you are a patient who is currently receiving outpatient care at our hospital, and your doctor believes that it is equally safe to attend to you in person (by physically coming to our center for each appointment) or remotely (via phone or video conference).

Study Objective

We aim to adapt to the modern times and utilize available technology to provide care to individuals without them having to leave their homes. However, it is essential to confirm the safety of this approach first, meaning that patients receiving care

remotely do not experience more medical issues or worsen compared to those receiving in-person care at the hospital. The objective of this study is to confirm that telephone or videoconference medical care is just as safe as the traditional in-person care.

Study Description

This study will involve 2136 patients receiving outpatient care at the hospital. All of them can be treated either remotely or in person, and it is not expected that the way they are treated will cause any problems.

The patients who agree to participate in the study will be randomly assigned (by chance) to receive in-person medical care (physically attending the appointment) or remote care (via telephone or video conference). Your doctor will record various aspects of your health's progress so that the researchers can confirm whether patients indeed fare equally well in all cases, regardless of the mode of care they received. This will allow us to use new technological methods of care with confidence and safety in the future.

Study Activities

If you agree to participate in the study by signing the informed consent form, you will be part of the study until you are discharged or until one year of follow-up is completed, whichever comes first.

Your participation in the study will only affect the way your doctor conducts visits (in person, by phone, or via video conference). Participation in this study will not change the number of visits, affect the tests your doctor requests, or influence the treatments you should take. All these aspects of your healthcare will be determined by your doctor based on what is most beneficial for your health, without any restrictions or conditions imposed by this study.

If you agree to participate in the study, your doctor will ask you at each visit whether you have been seen outside the hospital. The remaining data needed for the study will be filled in by your doctor after the consultation or extracted from the electronic medical record and the databases of the Catalan Health Service. Therefore, your

consultation with the doctor will not take longer than a couple of minutes if you decide to participate.

In addition to the above, a group of 100 participants will receive a phone call at the end of the study, during which an interviewer will ask questions about their satisfaction with the medical care received. Within this group, those who have used video calls to communicate with their doctor will also be asked about any difficulties they may have encountered with this technology. The interviewers involved in the study are hospital staff collaborating in the study and are subject to the same confidentiality rules regarding your data as the rest of the research team. In case you receive this call, you can be honest in your responses because the interviewers responsible for this part of the study will not disclose your answers to your doctor, nor will they record them in any place accessible to your doctor.

Risks and Discomforts Arising from Study Participation

Remote forms of assistance, whether by phone or via video conference, are commonly used to treat some patients in our hospital and throughout Catalonia. Therefore, your participation in the study does not entail any additional risks compared to the usual medical care at our facility. If you choose not to participate in the study, your doctor will decide the method of care (in person or remotely) based on the department's standard practice and your specific case. Therefore, you may receive remote care even if you do not want to participate in the study.

Please remember that your doctor has proposed your participation in this study because they believe that you can safely receive care through any of the three methods currently used in the hospital. Thus, your participation in the study only means that the method of care will be chosen randomly according to the research protocol, rather than being determined by your doctor based on the department's usual practice.

Potential Benefits

This study does not have direct benefits for you. However, by participating, you will help us confirm that the new forms of medical care, which are already widely used, are safe for patients like you.

Protección de datos personales

At any time, you can fully exercise your rights of access, modification, opposition, and cancellation of data, as well as limiting the processing of incorrect data and requesting a copy or transferring it to a third party (portability), as provided for in Regulation (EU) 2016/679 of the European Parliament and of the Council of April 27, 2016, on Data Protection (GDPR), as well as in Organic Law 3/2018 of December 5, 2018, on Personal Data Protection and Digital Rights Guarantee. You can exercise these rights by contacting your study doctor.

Please note that data cannot be deleted even if you stop participating in the study to ensure the validity of the research and compliance with legal obligations and medical product authorization requirements. You also have the right to contact the Data Protection Officer of CSAPG at the following email address: _________________@csapg.cat, and if you are not satisfied with the handling of your data, you can contact the Catalan Data Protection Authority.

The health data collected for the study will be identified by a code so that no information that can identify you is included, and only study collaborators will be able to link this data to you and your medical history. Researchers will also extract some data from the electronic medical record and the databases of the Catalan Health Service, which only personnel from the research team will access. Therefore, your identity will not be disclosed to anyone else except the health authorities when necessary or in cases of medical emergencies. The Research Ethics Committee, representatives of the Health Authority for inspection, and personnel authorized by CSAPG can only access your data to verify personal data, study clinical procedures, and compliance with Good Clinical Practice rules (always maintaining the confidentiality of the information).

The Investigator and the sponsor are obliged to retain the data collected for the study for at least 5 years after its completion. Subsequently, your personal information will only be retained by the center for your health care and by the sponsor for other scientific research purposes if you have given your consent to do so, and if permitted by applicable law and ethical requirements.

If we transfer your encoded data outside the EU to entities within our group, service providers, or scientific researchers collaborating with us, your data will be protected with safeguards such as contracts or other mechanisms stipulated by data protection authorities. If the participant wants to know more about this, they can contact the Data Protection Officer of CSAPG.

Expenses and Financial Compensation

This study does not offer any financial compensation for participants. It will not cost you anything either, as all study expenses are covered by the Consorci Sanitari de l'Alt Penedès i Garraf.

Study Discontinuation

As mentioned earlier, you can choose to stop participating in this study at any time, and it will not negatively impact your healthcare.

You should be aware that, exceptionally, you may be excluded from the study if the hospital authorities or study investigators deem it necessary, either for safety reasons, due to any change in your health that makes continuing the study inadvisable, or because they believe you are not complying with the established procedures. In any case, you will receive a proper explanation for the reason behind your withdrawal from the study, and this will not affect your future medical care.

By signing the attached informed consent form, you commit to complying with and facilitating the necessary procedures to carry out this study.

Contact for Questions

If you have any doubts or need more information during your participation, please contact the study doctor (contact information on the first page of this document).

You can also contact Dr. Alejandro Rodríguez Molinero, responsible for the Research Area and coordinator of this study (______@csapg.cat).

INFORMED CONSENT

STUDY TITLE	Study Protocol of a Randomized Controlled Trial to Assess Safety of Teleconsultation Compared with Face-to-Face Consultation. The ECASeT Study
STUDY CODE	CSAPG-15
SPONSOR	Consorci Sanitari de l'Alt Penedès i Garraf
INVESTIGATOR	
CENTER	
CONTACT	

I, ____

(participant's first and last name)

I have read the information sheet provided to me about the study.

I have been able to ask questions about the study.

I have received sufficient information about the study.

I have talked to____

(investigator's first and last name)

I understand that my participation is voluntary.

I understand that I can withdraw from the study:

- Whenever I want.
- Without having to provide explanations.
- Without this affecting my medical care.

I will receive a signed and dated copy of this informed consent document.

I freely give my consent to participate in the study and consent to the access and use of my data under the conditions detailed in the information sheet.

Patient's signature

Date: _____/____/_____

Investigator's signature

Date: ____/____/____

(Patient's and investigator's names, signature, and date in the patient's own handwriting)

Legal representative, family member, or de facto linked person

Date: ____/____/____

CONSENTIMIENTO INFORMADO

STUDY TITLE	Study Protocol of a Randomized Controlled Trial to Assess Safety of Teleconsultation Compared with Face-to-Face Consultation. The ECASeT Study
STUDY CODE	CSAPG-15
SPONSOR	Consorci Sanitari de l'Alt Penedès i Garraf
INVESTIGATOR	
CENTER	
CONTACT	

I, ____

(participant's first and last name)

I have read the information sheet provided to me about the study.

I have been able to ask questions about the study.

I have received sufficient information about the study.

I have talked to____

(investigator's first and last name)

I understand that my participation is voluntary.

I understand that I can withdraw from the study:

- Whenever I want.
- Without having to provide explanations.
- Without this affecting my medical care.

I will receive a signed and dated copy of this informed consent document.

I freely give my consent to participate in the study and consent to the access and use of my data under the conditions detailed in the information sheet.

Patient's signature
Date: _____/____/_____

Investigator's signature
Date: ____/____/____

(Patient's and investigator's names, signature, and date in the patient's own handwriting)

Legal representative, family member, or de facto linked person
Date: ____/____/____

(COPY FOR THE PATIENT)
